# Two Cases of Monkeypox-Associated Encephalomyelitis — Colorado and the District of Columbia, July–August 2022

**DOI:** 10.15585/mmwr.mm7138e1

**Published:** 2022-09-23

**Authors:** Daniel M. Pastula, Matthew J. Copeland, Markus C. Hannan, Samuel Rapaka, Takashi Kitani, Elizabeth Kleiner, Adrienne Showler, Cindy Yuen, Elizabeth M. Ferriman, Jennifer House, Shannon O’Brien, Alexis Burakoff, Bhavik Gupta, Kelli M. Money, Elizabeth Matthews, J. David Beckham, Lakshmi Chauhan, Amanda L. Piquet, Rebecca N. Kumar, Carlo S. Tornatore, Kia Padgett, Kevin O’Laughlin, Anil T. Mangla, Princy N. Kumar, Kenneth L. Tyler, Siobhán M. O’Connor

**Affiliations:** ^1^Neuro-Infectious Diseases Group, Department of Neurology and Division of Infectious Diseases, University of Colorado School of Medicine, Aurora, Colorado; ^2^Department of Infectious Diseases, Georgetown University Medical Center, Washington, D.C.; ^3^University of Colorado Health Memorial Hospital, Colorado Springs, Colorado; ^4^Department of Neurology, Georgetown University Medical Center, Washington, D.C.; ^5^Colorado Department of Public Health and Environment; ^6^Department of Pulmonology and Critical Care, Georgetown University Medical Center, Washington, D.C.; ^7^CDC Monkeypox Emergency Response Team; ^8^District of Columbia Department of Health, Washington, D.C.

*Monkeypox virus* (MPXV) is an orthopoxvirus in the *Poxviridae* family. The current multinational monkeypox outbreak has now spread to 96 countries that have not historically reported monkeypox, with most cases occurring among gay, bisexual, and other men who have sex with men (*1*,*2*). The first monkeypox case in the United States associated with this outbreak was identified in May 2022 in Massachusetts (*1*); monkeypox has now been reported in all 50 states, the District of Columbia (DC), and one U.S. territory. MPXV is transmitted by close contact with infected persons or animals; infection results in a febrile illness followed by a diffuse vesiculopustular rash and lymphadenopathy. However, illness in the MPXV current Clade II outbreak has differed: the febrile prodrome is frequently absent or mild, and the rash often involves genital, anal, or oral regions (*3*,*4*). Although neuroinvasive disease has been previously reported with MPXV infection (*5*,*6*), it appears to be rare. This report describes two cases of encephalomyelitis in patients with monkeypox disease that occurred during the current U.S. outbreak. Although neurologic complications of acute MPXV infections are rare, suspected cases should be reported to state, tribal, local, or territorial health departments to improve understanding of the range of clinical manifestations of and treatment options for MPXV infections during the current outbreak.

Details of two cases of encephalomyelitis associated with monkeypox in previously healthy young gay men in Colorado and DC are presented in this report. The University of Colorado and Georgetown University determined that this report was not subject to human subjects review because it includes only information obtained for purposes of patient clinical care and public health outbreak response. This activity was also reviewed by CDC and was conducted consistent with applicable federal law and CDC policy.^†^

## Patient A

**FIGURE 1:**
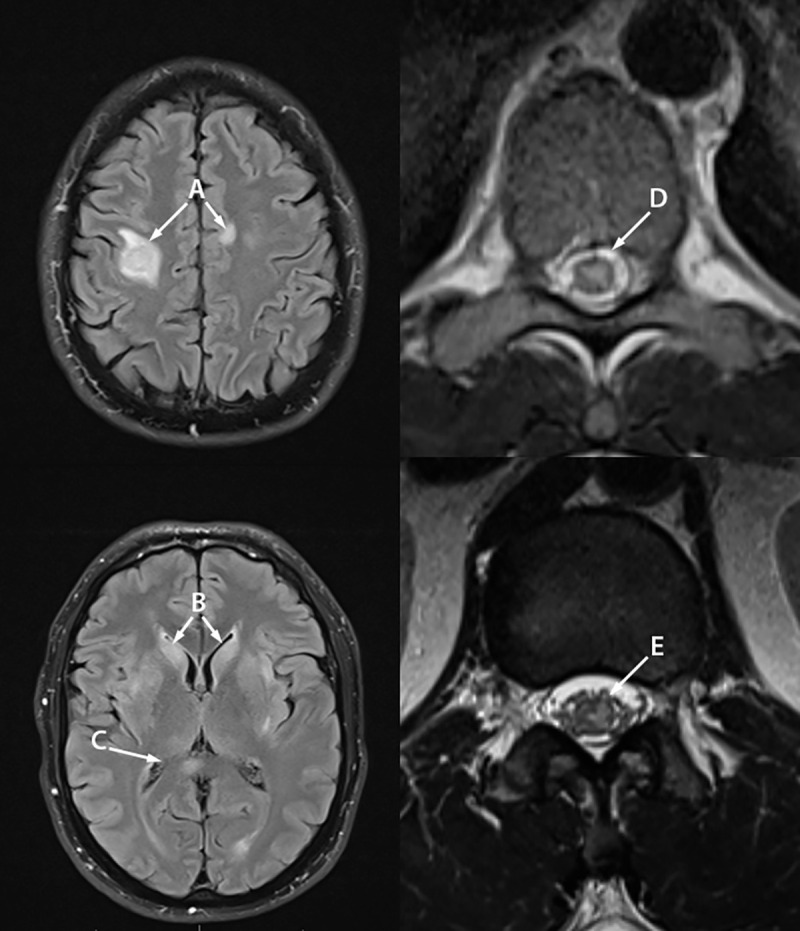
Magnetic resonance imaging of the brain, thoracic spine, and conus medullaris of patient A with monkeypox-associated encephalomyelitis showing abnormal T2/fluid attenuated inversion recovery signal in the right frontal and left frontal lobes (A), bilateral basal ganglia (B), bilateral medial thalami and right splenium (C), central thoracic spinal cord (D), and gray matter of the conus medullaris (E) — Colorado, July–August 2022 Photos/Daniel M. Pastula.

The first case occurred in a previously healthy, presumedly immunocompetent gay man in his 30s in Colorado (patient A). He had no recognized MPXV exposure or recent travel. He was not previously vaccinated against monkeypox or smallpox. In July 2022, he acutely developed fever, chills, and malaise. Three days after symptom onset, an itchy vesiculopustular rash appeared on his face and spread to his extremities and scrotum during the next several days. Swabs of a lesion yielded a positive polymerase chain reaction (PCR) test result for *Orthopoxvirus* DNA, later confirmed to be MPXV DNA. Nine days after symptom onset, the patient developed progressive left upper and lower extremity weakness and numbness, urinary retention, and intermittent priapism, and was hospitalized. Magnetic resonance imaging (MRI) of the brain showed partially enhancing lesions in the frontal lobes consistent with demyelination as well as nonenhancing lesions of the bilateral basal ganglia, bilateral medial thalami, splenium, and pons (Figure 1). MRI of the spine showed multifocal, longitudinally extensive, partially enhancing lesions of the central thoracic spinal cord and gray matter of the conus medullaris, with a single cervical level of canal stenosis with partial cord compression (presumably chronic and not acute). Cerebrospinal fluid (CSF) analysis demonstrated 155 white blood cells/*μ*L (normal = ≤5) with 60% lymphocytes, 30% monocytes, and 10% neutrophils; 9 red blood cells/*μ*L (normal = 0); glucose 64 mg/dL (normal = 45–80 mg/dL); and protein 273 mg/dL (normal = 15–45 mg/dL). CSF bacterial cultures were negative. CSF herpes simplex virus (HSV) and varicella zoster virus (VZV) PCR test results were negative. No CSF-specific oligoclonal bands (a marker for central nervous system [CNS] inflammation) were present. Serum aquaporin-4 (to evaluate for neuromyelitis optica spectrum disorder [NMOSD]^§^) and myelin oligodendrocyte glycoprotein (MOG) (to evaluate for MOG antibody–associated disease [MOGAD]^¶^) antibody test results were negative. Serum HIV serologic and PCR test results were negative. Serum treponemal antibodies and particle agglutination test results were positive; serum rapid plasma reagin (RPR) and CSF venereal disease research laboratory (VDRL) test results were negative, suggesting a past syphilis infection (patient A received a single dose of penicillin after an exposure in 2013). SARS-CoV-2 reverse transcription–PCR nasopharyngeal swab test result was negative, and serum and CSF MPXV PCR test results were negative.

Treatment with oral tecovirimat began immediately after the onset of neurologic symptoms. Subsequently, pulsed intravenous (IV) methylprednisolone (for suspected demyelination and spinal cord edema), IV immunoglobulin (IVIG) (for a possible parainfectious autoimmune process), and IV penicillin (for empiric syphilis treatment in case of a latent infection) were added to the patient’s regimen, with partial improvement in numbness and weakness over several days. After 2 weeks, the patient’s improvement plateaued with continued left leg weakness. Given concern for possible continued spinal cord inflammation, plasma exchange (PLEX) was initiated, and the patient’s leg weakness improved. His skin lesions resolved over 3 weeks. He was discharged to outpatient rehabilitation therapy and was ambulatory with an assistive walking device at 1 month follow-up. He was also referred to outpatient neurosurgery for his presumed chronic cervical spinal canal stenosis.

## Patient B

**FIGURE 2:**
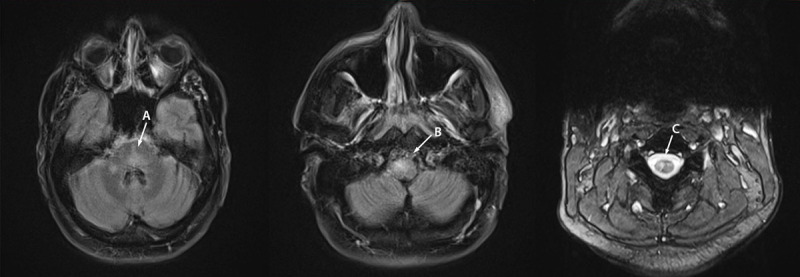
Magnetic resonance imaging of the brain and cervical spine of patient B with monkeypox-associated encephalomyelitis showing abnormal T2/fluid attenuated inversion recovery signal in the pons and cerebellum (A), medulla (B), and gray matter of the cervical spinal cord (C) — District of Columbia, July–August 2022 Photos/Matthew J. Copeland.

The second case of MPXV-associated encephalomyelitis occurred in a previously healthy, presumedly immunocompetent gay man in his 30s in DC (patient B). He had no known MPXV exposure or recent travel. He had not been vaccinated against monkeypox and his smallpox vaccination status was uncertain. In July 2022, he acutely developed fever and myalgia, which was followed by eruption of a diffuse vesiculopustular rash involving his face, extremities, trunk, and perianal area. Swabs of a lesion yielded positive *Orthopoxvirus* DNA PCR test results, later confirmed to be MPXV DNA. Five days after symptom onset, he developed bowel and bladder incontinence and progressive flaccid weakness of both lower extremities and was hospitalized. His condition progressed to altered mental status and obtundation during the next 2 days. He was intubated for airway protection and transferred to the intensive care unit. MRI of the brain showed nonenhancing lesions of the pons, cerebellum, and medulla without restricted diffusion (Figure 2). MRI of the spine showed multifocal, partially enhancing lesions in the central cervical and upper thoracic regions (Figure 2). Computed tomography imaging of the abdomen and pelvis demonstrated rectal thickening with pelvic lymphadenopathy consistent with proctitis, thought to be related to MPXV infection. CSF analysis demonstrated 30 white blood cells/*μ*L with 89% lymphocytes and 11% monocytes; 4 red blood cells/*μ*L, glucose 65 mg/dL, and protein 60 mg/dL. CSF bacterial cultures and CSF HSV and VZV PCR results were negative. Three CSF-specific oligoclonal bands were present. Serum and CSF aquaporin-4 and MOG antibody test results were negative. Serum HIV serologic and PCR test results were negative, as were serum RPR and CSF VDRL test results and rectal and urine gonorrhea and chlamydia screening results. SARS-CoV-2 reverse transcription–PCR nasopharyngeal swab test result was negative at admission and when febrile. CSF MPXV PCR test result was negative.

The patient started treatment with oral tecovirimat via nasogastric tube 2 days after neurologic symptom onset but quickly transitioned to IV tecovirimat over concerns for potential absorption issues. Because of concern for spinal cord edema, pulsed IV methylprednisolone was given with no immediate clinical improvement in weakness, but mild improvement in cognition. A parainfectious autoimmune process was considered, and IVIG was started. However, the patient subsequently developed high fevers, leading to discontinuation of IVIG after 2 days of treatment. A course of PLEX was initiated and the patient began to substantially improve. After five sessions of PLEX, he was extubated, was speaking and following commands, and had improvement in his lower extremity weakness. His proctitis resolved and his skin lesions healed by 5 weeks. He was given IV rituximab, a monoclonal antibody medication, for maintenance immunosuppressive therapy and was discharged to acute inpatient rehabilitation, ambulating with an assistive walking device.

## Discussion

Patients A and B had confirmed systemic MPXV infections with encephalomyelitis appearing within 5 and 9 days, respectively, of illness onset. The underlying pathology behind this is unclear but might represent either MPXV invasion of the CNS or a parainfectious autoimmune process triggered by systemic MPXV infection. Both patients had some clinical and radiographic features of acute disseminated encephalomyelitis (ADEM), typically a monophasic parainfectious autoimmune demyelinating disease of the CNS that primarily affects children but can also occur in adults (*7*). In past centuries, ADEM-like syndromes have been described in patients with presumed *Variola virus* infections (i.e., smallpox) (*8*,*9*).

In this report, neither patient was found to have MPXV nucleic acid in the CSF, which would have proven MPXV neuroinvasion. However, absence of detectable nucleic acid in the CSF is not uncommon among CNS viral infections. A CSF *Orthopoxvirus* immunoglobulin (Ig) M test for detection of virus-specific IgM antibodies, which could suggest viral neuroinvasion, was not performed because this test was not Clinical Laboratory Improvement Amendments (CLIA)–certified at the time of this report. Results of tests to look for the autoimmune CNS conditions NMOSD and MOGAD were negative. In patient A, neither the rash nor the neurologic condition was thought to be consistent with an active syphilis infection, and the cervical spinal canal stenosis did not fully explain his clinical condition.

Given that the pathologic mechanism for encephalomyelitis in these two instances is unknown, the best diagnostic workup and treatment course for similar cases is unclear. For severe MPXV disease, tecovirimat is recommended as first-line antiviral therapy, although the degree of CNS penetration is unknown (*10*). For significant edema, demyelination, or an ADEM-like presentation, corticosteroids can be considered, although benefits should be weighed against the immunosuppressive risks during an active infection. In addition, for a suspected parainfectious autoimmune CNS process or ADEM-like presentation, empiric IVIG or PLEX (or PLEX followed by IVIG) can be considered (*7*). The role for anti-B–cell therapies such as rituximab is not known.

Clinicians and public health professionals should be aware of the range of possible clinical presentations of MPXV infections and potential treatments. Suspected cases should be reported to state, tribal, local, or territorial health departments to improve understanding of the range of clinical manifestations of MPXV infections and treatment options. Persons who have been exposed to monkeypox or are at higher risk of being exposed may be vaccinated against monkeypox to reduce the chance of disease and can consider other protective measures to reduce their risk for exposure to MPXV.**

**Summary**

**What is already known about this topic?**

*Monkeypox virus* (MPXV) typically causes a febrile illness with lymphadenopathy and a diffuse vesiculopustular rash; neurologic complications are rare. The current monkeypox outbreak differs clinically and epidemiologically from previous outbreaks, and little is known about potential associated neurologic complications.

**What is added by this report?**

Two U.S. cases of encephalomyelitis associated with acute MPXV infection were identified during summer 2022. Whether the underlying pathophysiology resulted from direct viral neuroinvasion or a parainfectious autoimmune process is currently unknown.

**What are the implications for public health practice?**

Suspected cases of neurologic complications of monkeypox should be reported to state, tribal, local, or territorial health departments to improve understanding of the range of clinical manifestations of MPXV infections during the current outbreak and treatment options.

## Footnotes

† 45 C.F.R. part 46.102(l)(2); 21 C.F.R. part 56; 42 U.S.C. Sect. 241(d); 5 U.S.C. Sect. 552a; 44 U.S.C. Sect. 3501 et seq.

§ A chronic disorder of the brain and spinal cord dominated by inflammation of the optic nerve (optic neuritis) and inflammation of the spinal cord (myelitis).

¶ An inflammatory disorder of the central nervous system characterized by attacks of immune-mediated demyelination predominantly targeting the optic nerves, brain, and spinal cord.

** https://www.cdc.gov/poxvirus/monkeypox/prevention/index.html (Accessed September 7, 2022).
